# DBEndo: a web-based endodontic case management tool

**DOI:** 10.1186/s13104-015-1680-0

**Published:** 2015-11-17

**Authors:** Saskia Preissner, Eckehard Kostka, Mareike Mokross, Nina V. Kersten, Uwe Blunck, Robert Preissner

**Affiliations:** Department of Operative and Preventive Dentistry, Charité—Universitätsmedizin Berlin, Assmannshauser Straße 4-6, 14197 Berlin, Germany; Institute for Physiology and DKTK, Charité—Universitätsmedizin Berlin, Lindenberger Weg 80, 13125 Berlin, Germany

**Keywords:** Electronic health record, Electronic dental record, Database, Endodontics, Documentation, Statistics

## Abstract

**Background:**

The success of endodontic treatment depends—among many other factors—on good documentation. Paper-based records are often difficult to read or incomplete and commercially available tools focus on billing. An electronic record captures the state of treatment at all times. Databases are a common tool in everyday life.

**Results:**

Here, we present a database created for the Charité—Universitätsmedizin Berlin, Germany. Through consistent digital documentation, data analytics of patients, root canal anatomies, instrumentation techniques, efficacy of chemical disinfection, root filling techniques, and corresponding recall success rates, which needed extensive research before, are now easy to perform. Tables and even graphics and data analystics are only one click away and can be exported to other programs.

**Conclusions:**

DBEndo is a database to store and visualise internally, as well as to share endodontic cases online. For academic use we provide the database including all forms and some anonymous data for free at: http://dbendo.charite.de. Through easy import and export of the data, the system is open and flexible.

## Findings

### Background

Approximately 1125 root canals are treated each semester at the dental school of Charité—Universitätsmedizin Berlin. From time to time, dental students and/or dentists change during the course of root canal treatment (RCT). Paper-based records are often difficult to read or incomplete.

However, the success of endodontic treatment depends—among many other factors—on good documentation [1, 2]. This is very important if endodontic treatment is performed in more than one visit, because information on pain histories, reference points, X-ray measurements, irrigation protocols, canal preparations, intermediate dressings, or root canal obturation materials or techniques are essential. Different electronic dental record systems are commercially available—mainly focusing on billing issues, but the feasibility to use this data for research has already been studied. Databases have become a common tool in everyday life [3], several scientific journals publish database or server issues [4]. Most of the time, these databases are ‘special programs’, which require a lot of time for familiarisation. At the University of Pennsylvania the pennendo database was developed and interesting results are published frequently [5, 6]. Unfortunately, this database is only meant for internal research and is not purchasable or available for download. Meanwhile, there are techniques, which enable users to logon via web browsers. These solutions are very beneficial, if access is needed from more than one computer.

Documentation has to be fast and easy to use, so that the valuable time for treating patients is saved. The typing of text is time-consuming, so information entry should be via mouse clicks, touch screens or voice recording.

A recall-management system is important for seeing the success rates of RCT over the years. In daily routine, it is difficult to think of these recalls, so a reminder function for recalls within the database would be helpful.

### Database

The database is designed as a cross-platform relational database. This means that tables shelter data in sets and are connected with each other. To create the database, the software FileMaker ^®^ was used (FileMaker Pro Advanced 12.0 and Server Advanced Edition, FileMaker, Inc., Santa Clara, USA). The software provides user-friendly forms like calendars, lists, or drop-down menus. To make the input quick, easy and structured preselections for all fields were created and can be edited if needed. The database was optimised with several scripts for recall reminders, picture upload, and output of statistics through point-and-click.

### Web compatibility

For academic use, we provide the database including all forms and some anonymous data for free. The database can be used on a PC or Mac. Preselections can be edited, changes to the layout or content can be made for individual needs. It is possible to upload cases to share them with the community.

Some scripts and functions were adapted with the purpose of data protection. Different user profiles were generated and only one administrator is allowed to make changes within the database.

We created a user-friendly database that can be used by dentists in private practice or dental students. The focus was on simplicity so that the database can be used without any additional technical skills. Students use the database since 4 years. They are satisfied that they have structured data for their endodontic treatment within the database at any time.

### Usage of the database

For academic use, we provide DBEndo for free, at http://dbendo.charite.de/. On this page, a demo on how to use the database can be viewed by clicking on ‘Demo Tutorial’. To have a look at the database with some anonymous data, click on ‘Run Database’ and log in with the account name ‘user’ and the password ‘user’.

**Fig. 1:**
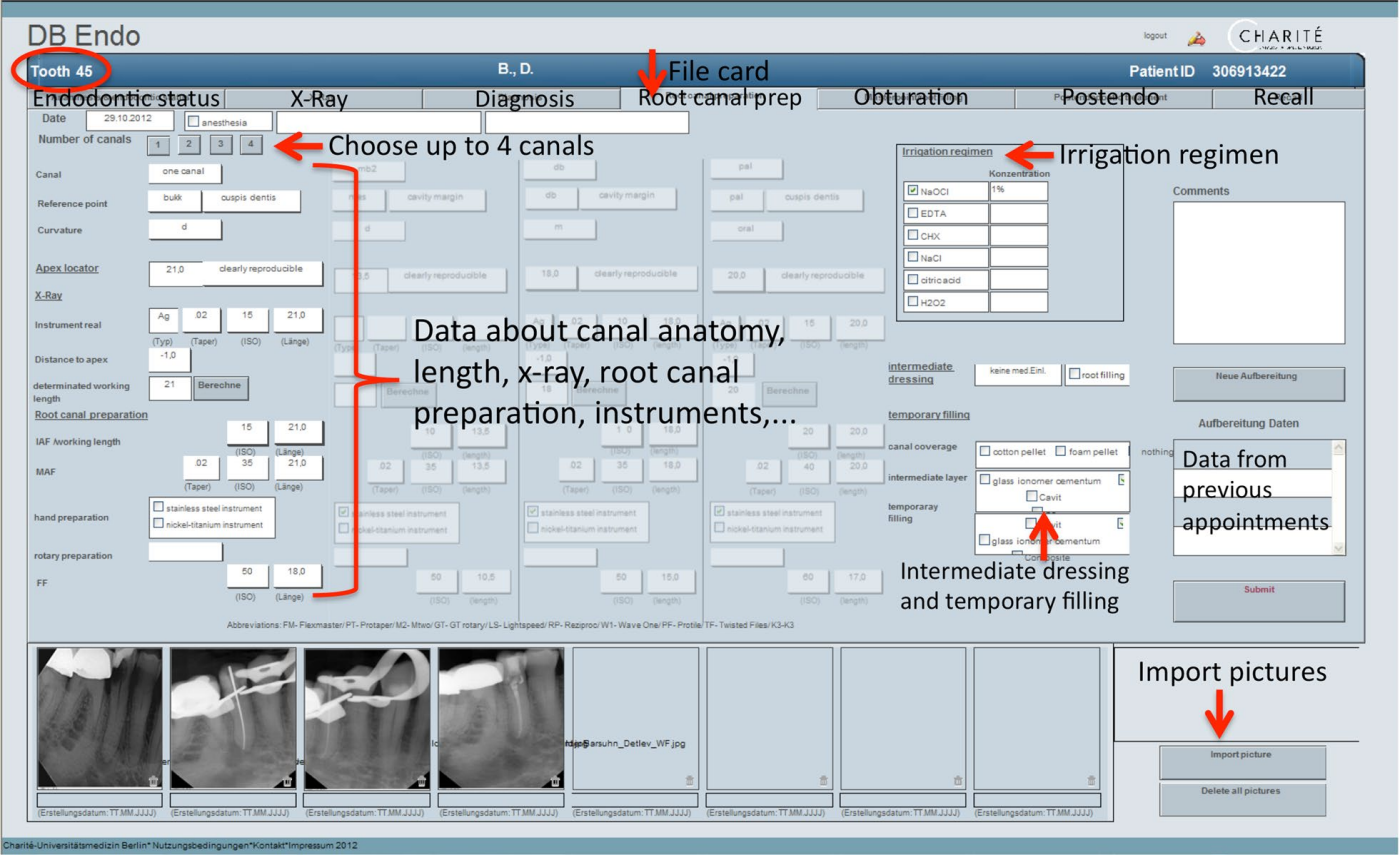
Main functionality of the database. File card ‘Root canal preparation’ is shown

The presentation layer of the database is ordered by file cards in the upper part leading users through the endodontic treatment (Fig. 1). There are seven file cards: anamnesis and endodontic status, X-ray, diagnosis, root canal preparation, master point/root filling, postendodontic treatment, and recall. In this figure, the main file card for root canal preparation is active. Up to four canals can be chosen and information on root canal anatomy, length, X-ray and instruments can be entered. On the right side of the file card, an irrigation protocol can be chosen and, if necessary, intermediate dressings can be selected. Data from previous appointments can be viewed and pictures (such as X-ray images or photos from a dental microscope) can be imported. A recall-management system with a reminder function facilitates the interpretability regarding success rates of RCT.

Cases can also be shared with the endodontic community. The database file including all forms is downloadable by clicking on ‘Download DB’. Once the database file is downloaded and Filemaker^®^ is installed, individual changes for the office or dental school can be made. To learn how to make changes within the database, a manual was created and can be found at ‘How To Modify’.

### Data analytics

To show the functionality of the DBEndo, RCTs of dental students from 2011/12 were used and anonymised. We included 300 patients between the age of 18 and 85 years, RCT beginning after 1st January 2011 and before 31st December 2011 by dental students and/or instructors, and patients with a physical address in Berlin/Brandenburg, Germany. The study protocol was approved by the Human Ethics Committee of the Medical Faculty of the Charité—Universitätsmedizin Berlin (EA1/327/13).

**Fig. 2:**
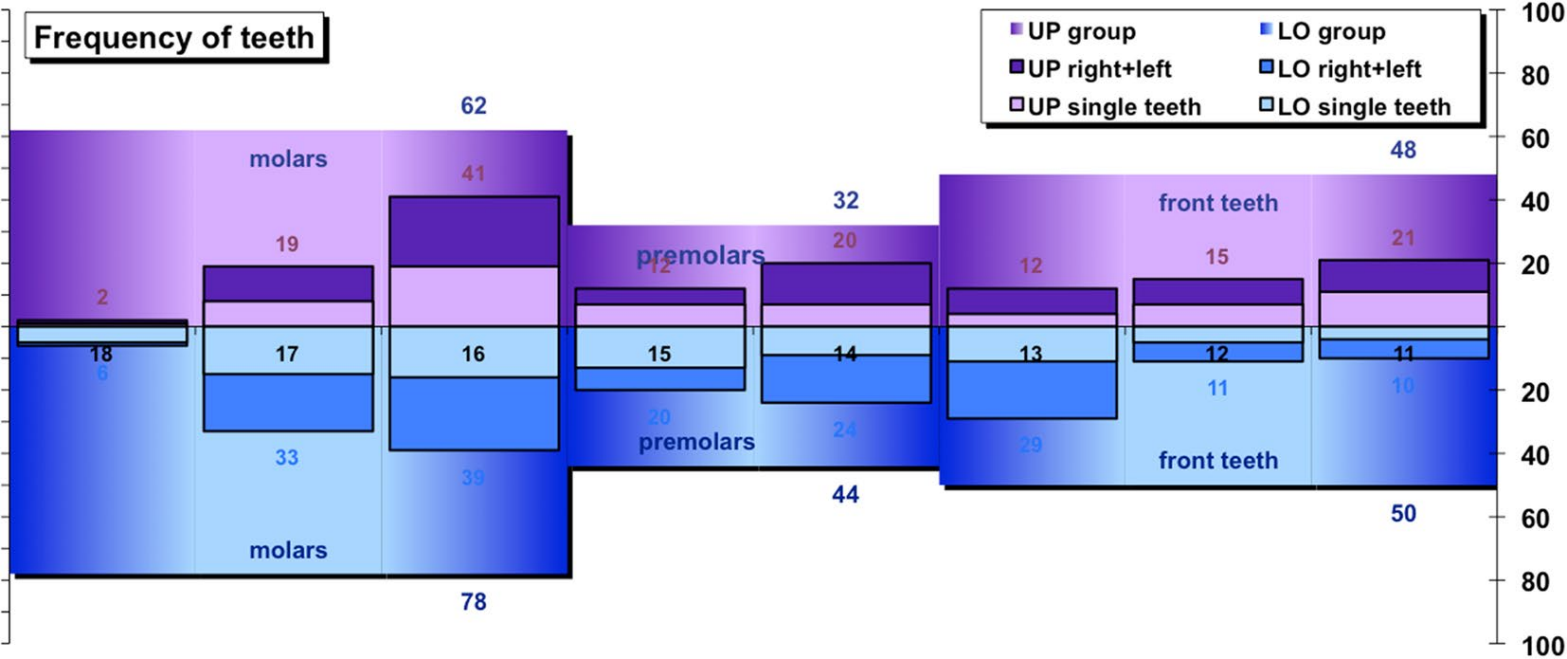
Frequency of root canal treated teeth

**Fig. 3:**
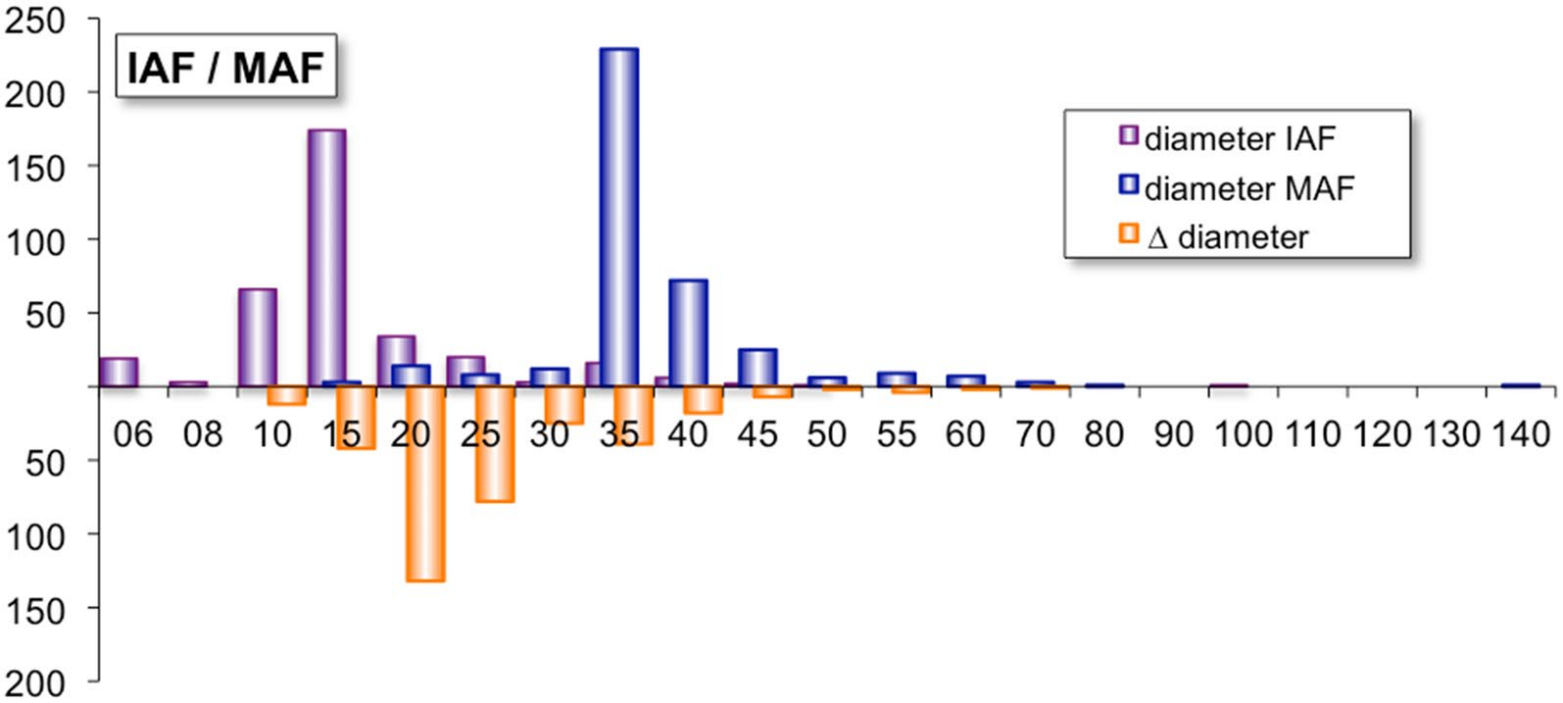
Correlation between initial apical file (IAF) and master apical file (MAF)

**Fig. 4:**
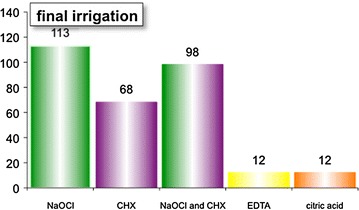
Overview of final irrigation solutions

**Fig. 5:**
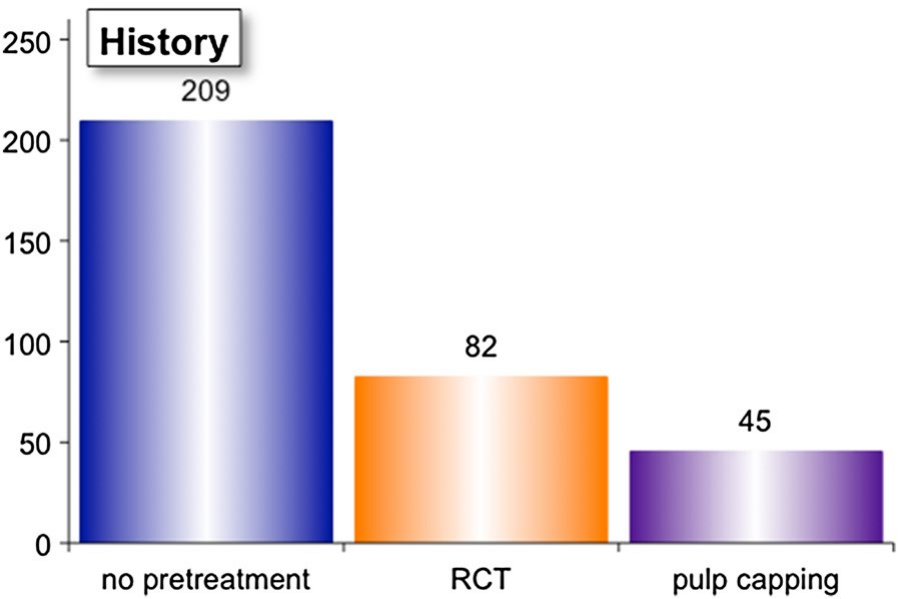
Overview of endodontic status at beginning of root canal treatment

The frequency of treatments per teeth (Fig. 2) can be viewed per tooth, per jaw or per group. The upper jaw (UP) is shown in purple, while the lower jaw is shown in blue. Teeth are grouped in molars, premolars, and front teeth. The enlargement during manual root canal preparation (Fig. 3) can be analysed by viewing the frequencies of the diameter of the initial apical file (IAF, shown in purple) and the diameter of the master apical file (MAF, shown in blue) are displayed. The apical enlargement is shown downwards in orange. The frequency of disinfection solutions used for final irrigation can be viewed (Fig. 4), as well as the number of teeth that had RCT or pulp capping in history (Fig. 5). Data can also be filtered by gender, vitality, diameter of apical radiolucency, periodontal conditions, reason for RCT, and many more.

### Conclusions

Handwritten documentation can be associated with poor legibility, which can contribute to medical errors [7]. Paper-based records require a great amount of storage space and have to be held for a minimum of 7–10 years. With paper-based records, it is difficult to collect information from different locations and to share records with multiple locations. electronic health records (EHRs) help with the standardization of forms, terminology, and data input. Data from EHRs can be used to share information over networks, track care and outcomes, trigger warnings and reminders, to perform statistics and research and to improve future treatment options. In Louisiana, an integrated EHR and public health information exchange system was implemented to automatically alert medical providers when a patient with HIV/AIDS had not received care in over 12 months, which reduced critical missed opportunities [8]. It has been shown that the EHR system improved the quality of diabetes care [9]. EHR software is often expensive, but there are several open source projects, such as OpenMRS [10]. This EHR platform grew out of the critical need to improve the treatment of HIV/AIDS in Africa and Haiti and to manage drug resistant tuberculosis in Peru. The system is built on the MySQL database, programmed in JAVA and can be modified without programming. In the United States, the Centers for Disease Control and Prevention reported that the EHR adoption rate had steadily risen to 44 percent, but fewer than half of US hospitals had a basic system in 2012 [11]. Estonia was the first country that has implemented a nationwide EHR system. Many other countries, such as Australia, Austria, Canada, Denmark, Netherlands, United Arab Emirates and Saudi Arabia initiated similar projects. The College of Dental Medicine in Florida uses EHRs since 2006 and recently reported, that EHR data can be a tool for strategic planning and oral health care programming [12].

DBEndo is a database to store and visualise internally as well as to share endodontic cases online. It provides well-adapted forms and is freely available for download for use in private practice. Changes can be made without programming. Through easy import and export of the data, the system is open and flexible. The database also has some limitations, such as the dilemma between structured data and enough flexibility for individual texts. Mandatory and free text fields were included to enable research, but to also make sure that there is enough space for individual remarks.

The database is planned to become an open source project for further development, as well.

## Availability and requirements

- Project name: DBEndo
- Project home page: http://dbendo.charite.de
- Operating system: platform independent
- Programming language: PHP/MySQL
- Other requirements: Javascript
- Licence: FileMaker Pro Advanced 12.0 and Server Advanced Edition
- Any restrictions to use by non-academics: licence needed.

## Abbreviations

DBEndo: Database Endodontics
IAF: initial apical file
MAF: master apical file
RCT: root canal treatment
EHR: electronic health record
OpenMRS: Open Medical Record System
MySQL: My Sequential Query Language
PHP: Hypertext Preprocessor
